# Utilizing whole genome sequences to study population genomics of gene networks: a case study of the *Arabidopsis thaliana* immune-signaling network

**DOI:** 10.1186/gb-2011-12-s1-p42

**Published:** 2011-09-19

**Authors:** Mridu Middha, Yungil Kim, Peter Morrell, Chad Myers, Fumiaki Katagiri

**Affiliations:** 1 Biomedical Informatics and Computational Biology, University of Minnesota, Minneapolis, MN 55455, USA; 2 Department of Plant Biology, Microbial and Plant Genomics Institute, University of Minnesota, St. Paul, MN 55108, USA; 3 Department of Computer Science & Engineering, University of Minnesota, Minneapolis, MN 55455, USA; 4 Department of Agronomy & Plant Genetics, University of Minnesota, St. Paul, MN 55108, USA

## 

*Arabidopsis thaliana* is a member of the mustard (Brassicaceae) family that is widely used as a model organism in plant biology. The 1001 Genomes Project [1] has been sequencing the genomes of *Arabidopsis* strains (accessions) and has made these sequences available. We selected the genomes of 30 *Arabidopsis* accessions with diverse geographical and environmental origins for our analysis. Using the TAIR8 annotation of the *Arabidopsis* reference genome, for the accession Col-0, we generated a dataset of approximately 27,000 protein-coding genes for all of the 30 genomes. With such population genomic data, it is feasible to ask whether a group of genes is under a different type of selection from the rest of the genome.

The plant immune-signaling network is robust to network perturbations. We hypothesized that genes that constitute a robust network tend to be under neutral selection because deleterious mutations in such genes do not strongly affect the immune phenotype owing to the robustness of the network. We identified the component genes of the plant immune-signaling network in a relatively unbiased manner by mining AraNet [2], which is a functional gene network built without using phenotype information. We compared population genetic summary statistics for the network component genes and those for all of the genes in the genome. For example, Tajima’s D is such a summary statistic, and positive, negative and zero values of Tajima’s D suggest diversifying, purifying and neutral selection, respectively, when the effective population size does not change. The Tajima’s D value distribution for all of the genes in the genome has a single clear peak with a negative value, suggesting that purifying selection is the genomic norm.

Our preliminary results showed that the plant immune-signaling network genes are significantly enriched with genes whose Tajima’s D values are near zero compared with all of the genes in the genome. This finding suggests that there is a lower level of purifying selection among the network component genes than other genes.
